# Factors modulating home range and resource use: a case study with Canarian houbara bustards

**DOI:** 10.1186/s40462-022-00346-1

**Published:** 2022-11-14

**Authors:** Inmaculada Abril-Colón, Juan Carlos Alonso, Carlos Palacín, Alberto Ucero, José Manuel Álvarez-Martínez

**Affiliations:** 1 Departamento de Ecología Evolutiva, Museo Nacional de Ciencias Naturales (CSIC), Madrid, Spain; 2 IHCantabria - Instituto de Hidráulica Ambiental de La Universidad de Cantabria, PCTCAN, C/Isabel Torres, 15, 39011 Santander, Spain

**Keywords:** Bird,3D-acceleration sensor, GSM/GPRS loggers, Habitat selection, Home range, Tracking

## Abstract

**Background:**

The home range of an animal is determined by its ecological requirements, and these may vary depending on many intrinsic and extrinsic factors, which are ultimately driven by food resources. Investigating the effects of these factors, and specifically how individuals use food resources within their home ranges is essential to understand the ecology and dynamics of animal populations, and to establish conservation measures in the case of endangered species. Here, we investigate these questions in the Canarian houbara bustard, an endangered subspecies of African houbara endemic to the Canary Islands.

**Methods:**

We analysed GPS locations of 43 houbaras in 2018–2021, using solar GSM/GPRS loggers provided with accelerometers. We assessed (1) the variation in their home range and core area with kernel density estimators in relation to several intrinsic and extrinsic factors and (2) their foraging habitat selection.

**Results:**

Home ranges were smallest during the breeding season (November–April), when rains triggered a rapid growth of herbaceous vegetation. Displaying males and nesting females had smaller home ranges than individuals not involved in reproduction. Both sexes used almost exclusively non-cultivated land, selecting low density *Launaea arborescens* shrublands, pastures and green fallows as foraging habitats. Heavier males used smaller home ranges because they spent more time displaying at a fixed display site, while heavier females moved over larger areas during the mating period, probably visiting more candidate mates. During the non-breeding season (May–October), both sexes showed larger home ranges, shifting to high density shrubland, but also partly to cultivated land. They selected sweet potato fields, green fallows, alfalfas, orchards and irrigated fields, which offered highly valuable food resources during the driest months of the year.

**Conclusions:**

Our study shows how Canarian houbara, originally a desert-dwelling species that uses mostly shrublands and pastures, has developed the necessary adaptations to benefit from resources provided by current low intensity farming practices in the study area. Maintaining appropriate habitat conditions in the eastern Canary islands should constitute a key conservation measure to prevent the extinction of this endangered houbara subspecies.

**Supplementary Information:**

The online version contains supplementary material available at 10.1186/s40462-022-00346-1.

## Background

The home ranges represents the space required by an animal to obtain resources necessary for survival and reproduction, and so defines the ecological requirements of a species [1–3]. Variation in home range is caused by several factors intrinsic and extrinsic to each population or species, such as density of individuals [4], habitat type and structure [5], body size [6], phase of the breeding cycle [7–9], and quantity and quality of food resources [10–12]. In a review of the functional relationships underlying the ecology of home range in birds, Rolando [13] found that food availability was the most important of eleven factors identified as relevant from the literature, and concluded that habitat selection was the most influential among four main processes determining home range size (i.e. habitat selection, breeding, mating, and flocking). Rolando’s [13] review highlights the importance of investigating which factors influence home range size, and also whether these same factors directly or indirectly affect foraging habitat selection patterns. These relationships between home range and foraging habitat selection are crucial to understand the ecology of animal species, and ultimately also their population structure and dynamics [14]. In order to establish habitat selection patterns that help us to better understand home range variation, it is necessary to know where individuals feed and what resources they use by studying fine-scale foraging locations, something that is only possible when behavioural data are available [15–19]. In a review about methods and most important questions in home range research, Fieberg [20] claimed that home range studies using behavioural data are very scarce, and recommended that more research should be done on this topic. In recent times, modern advances in tracking technologies have been increasingly used to study animal movement at high spatial and temporal resolution, helping us to understand the spatial ecology and habitat use of organisms [21–25]. However, few home range studies have benefited from these developments to relate foraging behaviour with habitat selection and home range use (see e.g., Zurell et al. [19]).

Finally, an aspect that may be decisive in determining home range and foraging habitat selection patterns in birds is whether the habitat is modified by agriculture. Over one third of all bird species use agricultural areas to some extent, particularly during the non-breeding season, and seeds, cultivated plants or weeds and animals associated to crops may represent a very relevant fraction of food resources for some species [26, 27]. It is therefore of high interest to determine to what extent the use of agricultural resources can influence the size of the home range and the selection of foraging habitat, particularly considering that croplands represent a large percentage of global land cover and are continuing to expand rapidly [28].

In this study, we used GSM/GPRS data loggers provided with GPS and accelerometers to investigate these relationships in Canarian houbara bustards (*Chlamydotis undulata fuertaventurae*), an endangered subspecies of African houbara endemic to the Canary Islands. This tracking technology allowed us to record very detailed data on individual home ranges, use of space, and foraging habitat selection. Previous studies had provided some data on home range and movements of houbara (*C. u. undulata*) and MacQueen’s bustards (*C. macquenii*) [29–37], but research on the endemic subspecies of the Canary Islands has only started very recently (Abril-Colón et al. [38]). This subspecies is restricted to the three easternmost islands of the Canarian archipelago: Lanzarote, Fuerteventura and La Graciosa. The population of Lanzarote, estimated at 440–452 individuals [39], represents the main stronghold, with about 80% of the total population [40–42]. This subspecies is classified as globally Vulnerable according to IUCN criteria [43], and a detailed knowledge of home range and foraging resources will help to understand how human pressures may affect its survival in an environment shared with millions of tourists visiting these small islands every year.

Our first objective was to analyze home range changes over the annual cycle in Canarian houbaras, in relation to the eight following intrinsic and extrinsic factors: (1) Sex. Home range may be expected to vary between sexes, given the sexual size dimorphism and sexual segregation in this species, the sexual differences in reproductive behaviour (territorial defense in males, chick rearing only by females), and different phenologies of territory occupation and migration [38]. (2) Season. Studies on home range seasonal variation are still scarce and have provided contradictory results, with some showing larger home ranges during the breeding season [44, 45] and others the opposite [46, 47]. In our case, we expected smaller home ranges during the breeding season in both sexes. (3) Reproductive status. Previous studies highlight its important effect on home range [48–50]. In the nominate subspecies of African houbara, females apparently visit various males to choose a mate and therefore need to move over a large area [32]. Thus, we expected females to have large home ranges during the mating phase, and small home ranges during the nesting period. In males, we expected displaying individuals to have smaller home ranges than non-displaying individuals. (4) Body weight and condition. Both have been found to influence home range in interspecific comparisons [2, 51, 52], and also within species [53]. In species where males defend territories, the physical condition is often related to territory size and quality [54, 55], with fitter males occupying better and often larger territories [56]. Thus, we hypothesized that houbaras with better body condition should have larger home ranges. (5) Body size. In many species, adult birds have longer wings than first-year or immature individuals [57–62]. In houbara bustards, as far as we know there are no data confirming a wing length increase with age, either in wild or in captive birds (Y. Hingrat, pers. com.), but first-year birds are known to usually retain outer primary feathers over the first year, and sometimes even up to an age of 24 months, and outer primaries are shorter and narrower in juvenile birds than in adults [63]. Therefore, it seems reasonable to assume that individuals with shortest wings in our sample were probably first- or second-year birds, and to expect that they should have larger home ranges during their juvenile-immature dispersal phase than adults.

Regarding extrinsic factors, we tested the following predicted relationships: (6) Density of individuals. In both sexes, we expected home range to be inversely related to population density due to density-dependence and competition with neighbours (e.g., [4, 53, 64, 65]. (7) Precipitation. Rainfall is strongly related with plant productivity. In arid environments, annual plants resume rapid growth after rainfall events [66] and plant productivity and diversity decrease after long-term reductions in water availability [67]. Therefore, we predicted that home range during dry months should be substantially larger than in rainy months. (8) Habitat quality. Many species have been found to use smaller home ranges in more productive areas [12, 68–70], or in areas with higher food availability [71–74], so we predicted that home range would be inversely related to plant productivity.

Our second objective was to investigate the use houbaras make of available food resources. Like in home range, we expected that habitat use should also differ between sexes, reproductive status and seasons, particularly in a semi-desert environment where resources are scarce and may represent an important limiting factor. Finally, in the case of endangered species, as in our study, exploring these effects of environmental and behavioural factors simultaneously on home range and resource use helps to establish clear space use patterns that may serve as a basis for meaningful conservation and management plans [23, 75, 76].

## Methods

### Study area and species

The study was carried out in Lanzarote (Canary Islands, 29°02’, 13°37’W; 986 km^2^). The Canary archipelago is located in the Atlantic Ocean, 140 km west of the northwestern coast of Africa. The climate is subtropical-desert, tempered by the cold Canary Current and the permanent northeasterly “Trade” winds. The rainfall is concentrated in December-February, with an average 110 mm per year. Summers are dry, with less than 1 mm precipitation in June–August. The island has a volcanic origin, and the vegetation is characterized by xerophytic shrubs, modified in some areas by goat grazing and farming activities. The mosaic of uncultivated and cultivated land in the centre of the island, with a combination of shrublands, fallows and sweet potato fields, most of them irrigated, facilitates weed growth and attracts houbara bustards in summer [38].

African houbara bustards are polygynous [77–79], and exhibit an exploded lek mating system [80]. They show a moderate male-biased sexual size dimorphism [81], own unpublished data for the Canarian subspecies). The endemic subspecies *C.u. fuertaventurae* is a nocturnal and partial migrant. Over one third of the individuals abandon their breeding areas and migrate to non-breeding areas with a mosaic of shrubland and cropland where they spend the hottest and driest months of the year [38]. Between late autumn and early spring, males of both, the nominate *C. u. undulata* and the insular subspecies concentrate at their lek areas where males display at specific locations of their territories, to which they generally remain faithful over the whole breeding period and also between years [38]. Females visit displaying males for mating and take over all breeding duties [82]. Successfully breeding females normally raise one, less frequently two or three chicks that remain dependent of her for several months. Females with dependent chicks abandon their breeding area later than unsuccessful females [38].

### Monitoring marked birds

Between 2017 and 2019, 43 houbaras (22 males and 21 females) were captured using nylon snares at display (males) or feeding sites (females). All birds were equipped with backpack-mounted, solar GSM/GPRS loggers (48 g model for males, 25 g model for females; e-obs GmbH, Gruenwald, Germany), using a soft, elastic band as harness material. The weight of transmitter plus harness was on average 2.83% of the body weight in males (range 2.54–3.15) and 2.15% in females (range 1.81–2.53). Males were captured at their display sites, which were selected randomly over the whole island, and females at foraging areas during the non-breeding season, in order not to jeopardize their nesting process. The capture team consisted of four people, who remained at 300–500 m from the capture site to be ready for access as soon as birds got entangled in the snares. Captured birds were immobilized, and their heads covered during the marking process to minimize capture stress. The average processing time of an individual from capture to release was 14 min (range 5–25). We did not observe behavioural alterations of the birds as a result of marking. Sex was established in the field using distinctive plumage features of females and males [83, 84], and confirmed by genetic analysis using DNA extracted from 1–2 contour feathers plucked from each bird.

The loggers recorded GPS locations between 05:00 and 22:59 UTC, i.e. from 1 (summer solstice) to 3 h (winter solstice) before sunrise to 1 to 3 h after sunset. All loggers were provided with an accelerometer (ACC) that registered the acceleration of the bird on a three-dimensional space, providing a 3D-graph representation of the bird's movements. We programmed the ACC between 05:00 and 21:00 UTC, obtaining 15-s activity bouts every 15 min, with a byte count of 1188 and 16.7 Hz. During the breeding season, we programmed the ACC of males during only 30 min (07:30–08:00 UTC), but using an intensive recording schedule of 45-s activity bouts every minute, because we were interested in recording in detail their display behaviour during the period of most intensive display at dawn for other studies (see more details below, in section *Habitat use and selection analyses*), and ACCs don't allow double programming within a day. The loggers were programmed to record one GPS location every five minutes when the charge level was high (95% of the time) and every 30 min otherwise. Of all ACC data recorded, we selected those coinciding with a GPS location, i.e. every 5 min, in order to be able to relate location with activity. Data were collected and stored in the Movebank repository (https://www.movebank.org/), and downloaded through the phone network, without having to recapture the birds.

### Data processing

A total of 3.2 million GPS locations from two years (2018, 2019) were used in this study. We distinguished two seasons in the annual cycle of houbaras, breeding and non-breeding [38]. Due to differences in body size and weight, ecological requirements, territory occupation phenology and migration dates of males and females (Abril-Colón et al. [38]; own unpubl. data), and because in the nominate subspecies both sexes exhibit different space and habitat use patterns [32, 80], we decided to analyse home range and resource use of each sex separately. In males, we defined as *Breeding season* the period they spent in their territories defending a display site. This period was delimited by the first and last displays (as a rule, respectively, early November–December and March–April). In females, the breeding season started with their first visit to the nesting area and finished on the last day of their nesting attempt (respectively, late November and April) [38]. In males that did not perform sexual display, we set the start and end of their breeding season on the average starting and ending dates of the display period of all males displaying on that year. Similarly, in non-nesting females we established the start of their breeding season on the average date when nesting females visited their nesting areas, and the end when all females breeding successfully had finished rearing their chicks. In both sexes, the rest of the year was considered *Non-breeding season*.

We analysed the effect of factors intrinsic and extrinsic to the individual on their home range size. As intrinsic factors we considered body size, body weight and reproductive status. For each bird we established a *Reproductive Status* based on the classification of Hingrat et al. [80]. During the breeding season we distinguished two reproductive statuses in males: 1) *Displaying*, when the male showed some sexual activity (vocalization, display run or pre-copulatory movements, as revealed by accelerometry), and 2) *Not displaying*, when the male didn't show any sexual activity over the whole breeding season, either because it was immature, or due to a suboptimal body condition on that particular year. In females, we distinguished four reproductive statuses: 1) *Mating*, when the female visited males during the mating period; 2) *Nesting*, when the female was incubating (incubation lasts about 23 days; [82, 85], (3) *Brooding*, when the female had 1–2 months-old dependent chicks; 4) *Not breeding*, when the female did not breed on that year. During the rest of the annual cycle (non-breeding season), all individuals were qualified with a *Non-reproductive* status. We used *Body weight* as an indicator of fat content and general body condition, instead of the more commonly used index of weight/tarsus length, because the tarsus was not measured in all individuals in order to minimise handling time [86]. As an index of *Body size* we used the wing length [60, 62, 87]. In many bird species, and specifically in those more related to houbaras like other bustards and cranes, larger males are usually older and/or dominant [58, 59, 88–91], thus wing length may be also a proxy for age and dominance status.

We also analysed the following extrinsic factors: *Precipitation* was obtained from the nearest of a total of seven meteorological stations available in Lanzarote island, located at an average distance of 2.13 ± 1.42 km. We obtained the *Density of individuals* from a census carried out in the breeding season of 2018 (census dates: 19 January-23 February; [39]. As for the non-breeding season, in order to minimize errors due to the higher mobility and lower faithfulness to a specific site during that period [38], we used the average values of two surveys, respectively 20 May–21 June 2018 and 22 May–11 June 2019. In order to obtain more representative density estimates, these were not restricted to just the area within the home range of our marked individuals,instead, we calculated them separately for eight regions: Zonzamas, Tahiche, Playa Blanca, Teseguite, Teguise, Costa Teguise, Playa Quemada and Soo. All houbara surveys were done by two teams each of two people driving vehicles at low speed through tracks and roads (more details in [39]. As a proxy of *Habitat quality*, we used the Soil-Adjusted Vegetation Index (SAVI), a vegetation index developed as a modification of the Normalized Difference Vegetation Index (NDVI) to correct for the influence of soil brightness when vegetative cover is low [92, 93]. SAVI is a more suitable indicator than NDVI in arid and semiarid environments like Lanzarote [94]. Autocorrelation is often cited as a problem associated with kernel density and other home range estimators (e.g., [95–98]. However, several authors have argued against sub-sampling of data [99, 100] and defend that the autocorrelation is not an issue for home range estimation, but has largely been a red herring, drawing attention away from the more important issue of obtaining a representative sample of locations [99, 101–103]. In studies based on telemetry, large samples are always better because they are more representative [102], and references therein).

### Home range analyses

Home range sizes were estimated using kernel density estimators (KDE) [104] and minimum convex polygons (MCP) [105, 106]. Kernel methods are the most statistically efficient nonparametric density estimators [107], since they do not make assumptions about the data distribution [108–111]. We used 50% kernels (KDE50) to delimit the core or most used areas, 95% kernels (KDE95) to define the total home range area, and MCP98 to represent the maximum area used by individuals, which was calculated including all outlier locations. We used the “reproducible home ranges” package (*rhr*) in R software for statistical computing [112, 113]. We established the reference bandwidth “href” as smoothing parameter for all individual home range estimations (see e.g., [114–116]). Although “href” may include areas outside an individual´s home range and thus may be positively biased, it shows a closer match between estimated and true home ranges with increasing sample size, so it is particularly recommended when sample size is large, as in our case [115]. We used monthly kernels for each bird as data points in the analyses, ignoring the tagging day and the two subsequent days to exclude any possible anomalous behaviour due to the capture and marking process. Periods of more than 15 days were considered as whole months, and those of less than 15 days were discarded. For each individual we used data from an average tracking period of 12.7 months (SD = 6.66, range 3–24). We also calculated MCPs, but did not use them in subsequent generalized linear mixed models (GLMMs). MCPs clearly overestimate true home ranges due to their lack of concavity and assumption of equal use of all locations [96].

We used the Wilcoxon signed-rank test to examine differences in home range size between seasons, and the Mann-Whitney test to examine differences between sexes and reproductive statuses, and Chi-squared test to examine foraging habitat selection. For each sex, we used GLMMs [117] to test variation in home range size over the annual cycle with the factors as described above: (1) season, (2) breeding status, (3) precipitation, (4) density of females, (5) density of males, (6) body size, (7) body weight, (8) habitat quality and (9) the interaction between season and precipitation. We considered this interaction because the effects of season on home range size could be expected to vary under different precipitation regimes, a frequent phenomenon in a semiarid area like the eastern Canary Islands. For example, we have detected a relationship between the onset of breeding and precipitation (pers. obs.). Since we had repeated measures of the same birds in two years, we included individual and year as random factors in GLMMs to avoid pseudoreplication. The response variable showed a negative binomial distribution and thus models were run using a loglink function. We used model averaging to calculate the predicted values. After computing parameter estimates averaged over all models of the dataset, we weighed them by using Akaike criterion at each model [118]. For all candidate models, we calculated the relevant parameters including the Akaike information criterion (AIC). Differences in AIC compared to the lowest AIC (ΔAIC), Akaike weight (W*i*), deviance explained and degrees of freedom were calculated, and the best model based on AIC was identified [119]. We obtained the parameter estimates and confidence intervals for each covariate included in all candidate models with > 5% of the weight of evidence (Additional file 1: Tables S2, S3). GLMMs were performed using the function *glmer* of the “lme4” package [120]. GLMMs were also performed to analyse variation in core areas. Since results were identical to those of home range analyses (see uploaded Related file), here we present only GLMM results for home range. The variables used did not show any collinearity problems. Mann-Whitney and Wilcoxon signed-rank tests were performed using the “wilcox.test function” and Chi-squared test with the “chisq.test function” in software R version 3.6.3 (https://www.r-project.org).

### Habitat use and selection analyses

In addition to exploring which variables explained the size of home ranges, we investigated which habitat types houbaras selected for foraging within their home range. We could carry out these analyses thanks to accelerometers (ACC), which enabled us to identify the ACC graph patterns and sequences that corresponded to the main activities of houbaras. To learn which graph pattern corresponded to each behaviour, we observed 10 marked birds from December 2018 to March 2019 (720 h in total) using a 20–60 × telescope from a distance of ca.1000 m. We recorded the timing of each behaviour and compared these timed field observations with the ACC graphs. We used the sequences of these behaviours to train a model using Accelerater, a software based on supervised machine learning, and translated ACC records to behavioural modes [121, 122], http://smell.huji.ac.il/). The model was trained on 2555 ground-truthed ACC sequences of known behaviours, and it classified ten behavioral modes: display run, pre-copulatory movement, vocalization, flying, foraging, laying down, pre-display posture, preening, running and vigilant posture. After assigning each GPS location to one of these behaviours identified from the ACC graphs, we filtered the subsample of foraging locations to analyze resource selection. Among various model types available in Accelerater, we obtained RBF SVM as the best one, with 92.95% correct classification (SD = 0.67), which means that all behaviours were identified with more than 90% accuracy.

As explained above, during the breeding season the ACCs of males were programmed to record acceleration data only from 07:30 to 08:00 h, so for breeding males we knew their behaviour only during that half-an-hour period. In order to estimate to what extent this could affect the foraging habitat use and selection results, we performed all calculations using both, this 30 min-ACC sample and the sample of GPS locations covering the period of maximum foraging activity (08:30 to 11:00 h), for which we had a GPS location every 5 min, but no behaviour associated to each GPS location. In other words, for that 2.5 h period we knew where each male was located, but not whether it was foraging, walking, or displaying. We did not appreciate any differences between both samples (07:30–08:00 h, and 08:30–11:00 h) and therefore we decided to use the 07:30–08:00 h locations in all subsequent analyses.

To analyse foraging habitat selection, we compared the GPS locations where each marked bird was feeding with a similar number of random points (pseudo-absences) generated within its home range. To prevent these pseudo-absences being on the same fields as foraging locations, we established a buffer of twenty metres for each foraging location and ensured that no pseudo-absence fell within those buffers. Since unbalanced prevalences may provide unstable and unreliable estimates of discriminatory power [123], a sampling effort was made to arrive at a balanced prevalence between presence (foraging locations) and pseudo-absence data (random locations). To increase the sample of breeding females and make it more similar to that of displaying males, in addition to the 6 females that bred in 2018–2019 we included breeding location data from 9 females in 2021.

We obtained the farming status and characteristics of all fields within the home range of each houbara from the 2020 Crop Production Map for Lanzarote (https://www.gobiernodecanarias.org/agricultura/temas/mapa_cultivos/lanzarote/), and completed or modified it with the 2018 orthophoto (http://www.grafcan.es) and direct observations of each field on the ground. This ground truthing was done by visiting all fields comprised within the home ranges of our marked birds and talking to as many owners as possible to determine the farming cycle phenology and other details of each field. The habitat types considered in this study were: Pastures; Shrubland of *Launaea arborescens*, where we distinguished two shrub density categories, high and low; Fallow, distinguishing a) Sweet potato fallow (where sweet potato was grown in the previous year), b) White fallow (ploughed continuously to prevent weed growth), c) Green fallow (no longer cultivated, weeds are left to grow); Alfalfa *Medicago sativa* fields, Orchards, Clean orchards (orchards where weeds are not allowed to grow), Sweet potato fields, and Sweet potato/fallow (mostly sweet potato crops with annual rotation of a cultivated sector and an uncultivated sector in the same field).

We compared the distributions of foraging and pseudo-absence locations of both sexes during the two seasons, breeding and non-breeding, using separate GLMMs for each sex and season. In order to get well-defined resource selection patterns for these two seasons, we used data from only the central months of the season when all birds of the sample were either breeding or had already started a post-breeding phase, i.e. December to February for the breeding season, and June to August for the non-breeding season. To avoid pseudoreplication we used data from only one year for each bird. We used GLMMs with binomial error structure to infer resource selection from use-availability data with presence *vs* pseudo-absence as response variable, individual as random factor, and habitat types (pastures, high or low density shrubland, sweet potato fallow, white fallow, green fallow, alfalfa, orchards, clean orchards, sweet potato and sweet potato/fallow-fields) as predictors. Presences and pseudo-absences for the resource selection analysis were plotted in ArcGIS, and software R version 3.6.3 (https://www.r-project.org) was used for all statistical analyses using the packages *rhr* [113], *MuMIn* [124] and *lme4* [120].

## Results

### Home range, core area and maximum area used

During the breeding season, the 19 displaying males and 6 breeding females used smaller home ranges (K95), core areas (K50), and maximum areas (MCP98) than the 6 males that did not display, and 18 females that did not breed (males: respectively, W = 13, *p* = 0.005; W = 13, *p* = 0.003; W = 15, *p* = 0.006; females: W = 14, *p* = 0.013; W = 13, *p* = 0.009; W = 14, *p* = 0.013; Mann-Whitney test; Table 1; see monthly values for both sexes in Additional file 1: Table S1). During the non-breeding season, the 19 males that had displayed used larger home ranges than during the breeding season (V = 16, *p* = 0.002 in K95; V = 8.5, *p* < 0.001 in K50; V = 29, *p* = 0.008 in MCP98; Wilcoxon signed-rank test). The same trend was observed in the 6 females that had bred, although female differences did not reach significance, probably due to the small sample size (V = 3, *p* = 0.625 in K95; V = 1, *p* = 0.250 in K50; V = 4, *p* = 0.875 in MCP98; Wilcoxon signed-rank test). During the non-breeding season, the home ranges of the 19 males that had displayed and those of the 6 females that had bred did not significantly differ in size from the home ranges used during the breeding season by the 6 males that did not display (W = 44.5, *p* = 0.859 in K95, W = 63, *p* = 0.862 in K50: W = 46.5, *p* = 0.524 in MCP98) and the 18 females that did not breed (W = 24, *p* = 0.721 in K95, W = 27, *p* = 0.487 in K50 and W = 27, *p* = 0.957 MCP98; Mann-Whitney test; Table 1).

**Table 1:** Mean home-range (KD95), core area (KD50) and maximum area used (MCP98) by male and female houbara bustards depending on reproductive status and season

	Displaying n = 19^1^	Not displaying n = 6^1^	Breeding n = 6^1^	Not breeding n = 18^1^
	Males		Females	
Breeding season				
K95	0.61 ± 0.43 (0.19 − 1.93)	2.61 ± 1.52 (0.37 − 5.34)	0.89 ± 0.60 (0.14 − 8.10)	2.13 ± 1.17 (0.37 − 7.57)
K50	0.08 ± 0.05 (0.02 − 0.19)	0.48 ± 0.32 (0.05 − 0.99)	0.16 ± 0.12 (0.02 − 3.12)	0.58 ± 0.56 (0.05 − 1.91)
MCP98	1.06 ± 0.78 (0.27 − 3.57)	4.40 ± 2.60 (0.58 − 8.58)	1.31 ± 1.03 (0.39 − 10.59)	2.84 ± 1.61 (1.06 − 8.69)
	Males (n = 22)		Females (n = 21)	
Non-breeding season				
K95	1.12 ± 0.61 (0.25 − 2.60)		1.43 ± 1.18 (0.23 − 9.05)	
K50	0.20 ± 0.10 (0.04 − 0.44)		0.28 ± 0.30 (0.04 − 1.25)	
MCP98	1.98 ± 1.57 (0.37 − 6.59)		2.72 ± 2.55 (0.27 ± 9.81)	

Values given are means (km^2^) ± SD, and range (min–max) for both study years

^1^Total samples were 22 males and 21 females, but 3 males and 3 females changed their reproductive status between both study years and therefore were included in both subtotals

As for sexual differences, during the breeding season there were no differences in home range size between displaying males and breeding females (W = 68, *p* = 0.224 in K95; W = 60, *p* = 0.113 in K50; W = 79.5, *p* = 0.491 in MCP98), nor between non-displaying males and non-breeding females (W = 32.5, *p* = 0.555 in K95, W = 26, *p* = 0.953 in K50, W = 37, *p* = 0.2625 in MCP98; Mann-Whitney test; Table 1). During the non-breeding season, there were no sexual differences in home range size (W = 213, *p* = 0.885 in K95; W = 183, *p* = 0.693 in K50; W = 181.5, *p* = 0.480 MCP98; Mann–Whitney test; Table 1). The mean overlap between post-breeding areas in different years was 61.6% in males and 47.2% in females. As for breeding areas, the mean overlap was 52.1% in males and 42.2% in females (Additional file 1: Fig. S1).

Although the bivariate analysis did not show significant sexual differences within a given season, we performed generalized linear mixed models separately for males and females, because both sexes exhibit various contrasting features during their annual cycles (e.g. different territory occupation and migration phenologies, Abril-Colón et al. [38]), and certain factors could have distinct effects on male and female home ranges. Thus for each sex we obtained multiple models to describe home range (Tables 2, 3, details in Additional file 1: Tables S2, S3), of which for the sake of brevity and clarity here we make inferences only from those selected though Akaike criteria as the most plausible ones. The best model explaining home range in males included season, precipitation, reproductive status, body weight and habitat quality, plus the interaction between season and precipitation (Table 2). A second valid model included also the effect of body size. Sums of the AIC weights showed season, reproductive status, precipitation and body weight to be the best predictors of home-range size (*w*_*i*_ = 1). These models confirmed the variation of home range with season and with reproductive status, with non-displaying individuals using larger areas than displaying individuals (Additional file 1: Table S2). In addition, home ranges were larger when precipitation, body weight and habitat quality were lower (Additional file 1: Table S2). Also, a positive interaction between season and precipitation was found.

**Table 2:** Candidate generalized linear mixed models analysing the effect of intrinsic and extrinsic factors on the home range size (K95) of males (n = 22)

	AIC	ΔAIC	W*i*	Explained deviance	*df*
S + P + S*P + RS + BW + HQ	9675.19		0.431	25.70	9
S + P + S*P + RS + BS + BW + HQ	9676.83	1.64	0.178	25.77	10
S + P + S*P + RS + BW	9678.04	2.85	0.110	24.69	8
S + P + S*P + RS + DF + DM + BS + BW + HQ	9678.25	3.06	0.081	26.30	12
S + P + S*P + RS + DM + BS + BW + HQ	9678.26	3.07	0.075	25.89	11
S + P + S*P + RS + DF + BS + BW + HQ	9678.55	3.36	0.070	25.83	11
S + P + S*P + RS + DF + BW	9679.33	4.14	0.054	24.83	9

We analysed the effect of season (S), precipitation (P), reproductive status (RS), body size (BS), body weight (BW), habitat quality (HQ), density of females (DF), density of males (DM), and the interaction between S and P (S * P). Summary statistics include Akaike information criterion (AIC), difference in AIC (ΔAIC), Akaike weight (Wi), deviance explained and degrees of freedom (df). Models are ranked from best to worst according to AIC

**Table 3:** Candidate generalized linear mixed models analysing the effect of intrinsic and extrinsic factors on the home range size (K95) in female houbara bustards (n = 21)

	AIC	ΔAIC	W*i*	Explained deviance	*df*
RS + P + DM + BS + BW + HQ	569.96		0.233	32.14	11
RS + P + DF + BS + BW + HQ	569.99	0.03	0.230	32.16	11
RS + P + DF + DM + BS + BW + HQ	570.77	0.81	0.156	32.64	12
S + RS + P + DM + BS + BW + HQ	570.97	1.01	0.141	32.54	12
S + RS + P + DF + BS + BW + HQ	571.71	1.75	0.097	32.27	12
S + RS + P + DF + DM + BS + BW + HQ	572.14	2.18	0.079	32.90	13
RS + DF + BS + BW + HQ	573.65	3.69	0.037	29.87	10
S + P + DF + BS + BW + HQ	574.33	4.37	0.026	27.76	8

We analysed the effect of season (S), reproductive status (RS), precipitation (P), density of females (DF), density of males (DM), body size (BS), body weight (BW), and habitat quality (HQ). Summary statistics include Akaike information criterion (AIC), difference in AIC (ΔAIC), Akaike weight (Wi), deviance explained and degrees of freedom (df). Models are ranked from best to worst according to AIC

As for home ranges of females, the first two plausible models showed almost identical weights, so both may be considered equally valid (Table 3). These models included the effects of reproductive status, precipitation, density of males (first model only) or females (second model only), body size, body weight and habitat quality. A third, also valid model (ΔAIC < 2) included both, density of males and density of females. The sums of AIC weights showed reproductive status, precipitation, habitat quality, body size, body weight, density of males and density of females to be the best predictors of home range size (w_i_ > 0.656). As in males, reproductive status had in females a significant and even higher effect than in males, with nesting females showing the smallest areas, non-breeding females the largest, and mating and brooding intermediate values (Table 3, Additional file 1: Table S3, Additional file 1: Fig. S2). In females, home range increased with precipitation and habitat quality, in contrast to what happens in males. Female home range also increased when body size was smaller, and when density of females and males (first model and second model, Table 3) (were lower (Additional file 1: Table S3). Finally, the fourth and fifth candidate models included season, a variable that was not retained in the first three models (Table 3). These fourth and fifth models showed ΔAIC < 2 and therefore could also be considered plausible to some extent, but as shown in the bivariate analysis, the effect of season was apparently less pronounced in females than in males.

### Selection of foraging habitat types

In both sexes, the distribution of foraging locations among habitat types differed from that of locations not used for foraging (males: *X*^2^ = 6568.68, df = 10, *p* < 0.001 in the breeding season; *X*^2^ = 27,179.15, df = 10, *p* < 0.001 in the non-breeding season; females: *X*^2^ = 1158.17, df = 10, *p* < 0.001 in breeding season, *X*^2^ = 4137.94, df = 10, *p* < 0.001 in the non-breeding season; Chi-square test). During the breeding season, displaying males and breeding females foraged almost exclusively on non-cultivated land, using primarily low-density *Launaea* shrubland and pastures (Additional file 1: Table S4). The only two non-displaying males and four females of our sample that did not breed used pastures as their main foraging habitat, followed in the case of females by sweet potato, green and sweet potato fallows, and low-density shrubland (Additional file 1: Table S4).

During the non-breeding season, the use of cultivated land increased in both sexes, reaching 26.58% of all foraging locations in females, and 17.73% in males. This seasonal increase in the use of cultivated fields was significant in both sexes (*X*^2^ = 1069.2, df = 1, *p* < 0.001 in males, *X*^2^ = 4626.9, df = 1, *p* < 0.001 in females; Chi-square test; Additional file 1: Table S4). A significant majority of these cultivated fields were irrigated (*X*^2^ = 1110, df = 1, *p* < 0.001, *X*^2^ = 365.3, df = 1, *p* < 0.001; Chi-square test, comparing locations in irrigated vs non-irrigated fields respectively in males and females; Additional file 1: Table S4). In this season both sexes continued showing a preference for *Launaea* shrubland as the main foraging ground, though males used higher density shrubland more than lower-density shrubland. Females spent more time foraging on sweet potato fields, green fallows and alfalfa fields than in the breeding season, and used these habitats more than males (Additional file 1: Table S4).

The results of GLMMs showed that houbara bustards chose foraging sites that were significantly different from randomly selected pseudo-absence locations. We found noteworthy seasonal differences and some sexual differences in foraging habitat selection. In the breeding season, displaying males selected low-density shrubland and green fallows as main foraging habitats, avoiding shrubland with high density coverage, sweet potato fallow and white fallows, while in the non-breeding season they showed a preference for high-density shrubland, sweet potato fields and orchards (Table 4; Additional file 1: Tables S5 and S6). As for females, during the breeding season they selected pastures, green and white fallows and sweet potato/fallow (Table 4; Additional file 1: Table S7). During the non-breeding season females showed similar selection patterns as males, avoiding pastures, white fallows, clean orchards and sweet potato/fallow, and selecting high-density shrubland and green fallows, together with sweet potato fields (Table 4; Additional file 1: Table S8).

**Table 4:** Estimates of generalized linear mixed models describing fine-scale foraging habitat of male and female houbara bustards during both seasons

Predictors	Males^1^		Females^1^	
	Breeding season	Non-breeding season	Breeding season	Non-breeding season
	Displaying		Breeding	
Pastures	− 2.389***	− 1.334***	0.554***	− 0.179**
High density shrubland	− 3.079***	2.245***	− 0.934***	0.315***
Low density shrubland	1.177***	− 0.254	− 0.326***	0.029
Sweet potato fallow	− 4.242***	24.007	22.160	− 1.494***
White fallow	− 0.598^(*)^	− 23.872	2.411***	− 4.306***
Green fallow	2.506***	0.271	1.118***	0.266***
Alfalfa	–	–	–	23.048
Orchards	3.072	0.467**	− 20.73	− 2.269***
Clean orchards	− 9.591	− 2.756***	− 34.94	− 0.771***
Sweet potato	− 3.864	1.512***	33.11	1.150***
Sweet potato/fallow ^2^	− 3.947	− 0.152	0.536*	− 1.083***
Explained deviance	25.56	22.38	24.87	19.64

GLMMs were fitted using binomial error structure and individual as random factors. GPS data were filtered to include only foraging locations

*P*-values: ***p < 0.001, **p < 0.01, *p < 0.05, ^(*)^ *p* < 0.1

^**1**^Due to the small sample size and low explained deviance, the GLMM for non-displaying males and not breeding females is not included in Additional file 1. ^2^ Mostly sweet potato crops with annual rotation of a cultivated sector and an uncultivated sector of the same field

## Discussion

Our results show that much of the variation in home range and foraging habitat selection was determined by season and reproductive status. During the breeding season, displaying males and nesting females had much smaller home ranges than males and females not involved in reproduction. Among non-breeding individuals with large home ranges, our sample surely included some immature birds, as suggested by the negative relationship between home range and wing length found in models. This relationship was significant in females and also negative though not significant in males, in which the only size-related variable retained in the best model was body weight, also with negative effect on home range. These results agree with the large areas used by dispersing juveniles in the nominate houbara subspecies [125], as well as with the common pattern found in other bird species, where non-breeding adults, floaters, or dispersing immatures show larger home ranges than breeding individuals [18, 19, 126, 127]; see review in [13].

The small home ranges found in displaying males and the largest home ranges of mating females are consistent with the *exploded lek* mating system attributed to this species [31, 32, 37, 38, 80]. Within the lek area, male houbaras defend small territories where they display over several months [128] They remain faithful to these territories throughout the whole breeding season and between years [38, 129]. As for breeding females, their home ranges are large during the mating phase, when they typically visit several males [32] and even mate with more than one as suggested by the high proportion of broods with multipaternity [130]. The positive effect of body weight on home range found only in females suggests that females moving over larger areas and visiting more males are those in better condition. In males, home range is negatively related to body weight, because males in better condition spend more time displaying and therefore move less during that period than lighter, non-displaying males. Individual and seasonal variation in weight is higher in males than in females ([131–133], own unpubl. data for the Canarian subspecies). Males gain 15–20% weight to reach highest weights at the start of the breeding season, losing weight considerably during the display period when they spend little time feeding [37, 128]). Heavier males display more intensively and weight loss during display is correlated with display intensity, so weight represents a signal of male quality [131, 134–136], explaining the negative relationship between weight and home range found in our models. Differences related to reproductive status disappeared during the non-breeding season, when home ranges of individuals that had been involved in breeding activities equaled those of non-breeding and immature individuals, and all birds showed larger home ranges than during the breeding season.

Precipitation and habitat quality also showed opposite effects in males and females. Although monthly values of these two variables were not highly correlated and thus we included both in home range models, to some extent they have similar meanings in relation to reproduction in houbara bustards. Both had a positive effect on home range in female models and a negative effect in male models. Precipitation has a strong influence on circannual rhythms of species inhabiting arid environments [137–141], and these effects have been also documented in houbara bustards. For example, breeding is suppressed in dry years, and rainfall seems to trigger the onset of breeding in this species ([142–144], own unpubl. data), possibly through mechanisms inducing gonadal activity [133]. As for SAVI, it has been commonly used as a proxy for net primary production in poorly vegetated environments such as our study area [94]. As expected, monthly values of both precipitation and habitat quality had similar effects on the breeding phenology of houbaras because autumn and winter rainfalls produce a rapid growth of herbaceous vegetation, inducing males to start displaying and females to begin searching for a mate. This happens in less than a month, explaining why both variables produce the same effect within each sex but opposite effects on male and female monthly home range models. Shortly after the first rainfalls males occupy their territories and reduce their home range to a minimum, whereas females increase it while searching for mates.

With respect to differences in foraging habitat related to season and reproductive status, we found that during the breeding season both sexes showed a preference either for low density shrubland or pastures, though the most selected of these habitats differed between sexes, males preferring shrubland and females pasture land. This sexual difference might only be due to the fact that males use high points to display, and these are usually occupied by shrubland, whereas pastures are mostly on valleys. In any case, both habitat types really differ from each other only very slightly, representing quite areas where both sexes can carry out their breeding activities without being disturbed. Both offer similar amounts and diversities of weeds and invertebrates consumed by breeding houbaras [145, 146]. A third, much less used but still selected foraging habitat were green fallows, where weeds are allowed to grow and thus offer similar food as pastures or shrublands. During this season, both sexes avoided foraging in high density shrubland, probably because in this habitat visibility is hindered by bushes. A good visibility is crucial for displaying males [37, 129, 147] and for nesting females of the nominate subspecies, which avoid areas with tall vegetation [37]. In great bustards, males and females also select display and nesting sites with good visibility, probably to attract more females in the case of males, and to reduce predation pressure in both sexes [148, 149]. In houbaras, concealment provided while foraging by low density shrubland might be appropriate for nesting and brooding females, but a too high shrub density might not be appropriate.

During the non-breeding season foraging habitat models showed a shift to high density shrubland where birds find quiet and hidden sites for feeding in summer, no significant selection for low density shrubland, and negative selection for pastures. During this season, birds of both sexes increased significantly the use of cultivated land, particularly sweet potato fields, and selected irrigated over non-irrigated fields. A previous study found that in the island of Fuerteventura houbaras selected *gavias* as foraging grounds, a traditional mode of cultivation designed to retain rain and runoff water equivalent to modern irrigation systems [146]. Although natural grasslands are originally the main habitat of the family Otididae [81], several species forage on farmland, and increase the use of cultivated fields during the non-breeding season, e.g. the Great Bustard (*Otis tarda*) ([150–154]), the Little Bustard (*Tetrax tetrax*) [155, 156], or the MacQueeen's bustard [157]. Cultivated areas represent an additional food resource for many other birds, which have adapted to human-induced landscape changes, and in some cases have become agriculture specialists [158, 159]).

Regarding sexual variation, in contrast to what we expected, we found no differences between males and females in their average home ranges during either the breeding or non-breeding season. Neither we found important sexual differences in foraging habitat selection. Essentially, both sexes had the same requirements regarding home range and food resources. It seems that sexual dimorphism, sexual segregation, and differences in roles during reproduction and in phenologies of migration and display and nesting territory occupation are not enough to cause great sexual differences in average home range size or foraging habitat, either in the breeding or non-breeding season. However, sexual differences in behaviour could have indeed determined the different sign of the relationship between home range and some of the variables in our models. For example, an increasing density of conspecifics didn't seem to affect much the home range of males, whereas it determined a significant reduction of home range in females. A density-dependent restriction on home range size is expected when breeding habitat is limited [12, 64, 160–163]. Density-dependent effects on home range may probably be small during the non-breeding season, when houbaras can aggregate in small flocks of up to 5–6 individuals, but may affect territories of nesting and brooding females due to competition with neighbour females or sexual harassment when male density is high. The size of male territories, in contrast, didn't seem to be as density-dependent as that of females. A male territory may be restricted to the minimum area necessary to perform display and mating activities without being disturbed by neighbour males, and thus probably does not extend farther even under low conspecific densities, simply because a larger territory is not economically defendable. In support of this conclusion, the variability and maximum extent of home ranges were smaller in displaying males than in breeding females. In sum, home range sizes of males and females seem to fit with the exploded lek mating system attributed to houbara bustards. While in classical lek species female home ranges are larger than those of males, in resource-defence polygyny systems both sexes show no differences in home range size, and exploded leks occupy an intermediate position in this gradient [164–166].

Beyond the small sexual differences in density-dependence discussed above, home ranges and core areas of Canarian houbaras are much smaller than those reported for the nominate subspecies in north Africa (home ranges of 17 km^2^ in males and 146 km^2^ in females, core areas of about 1 km^2^; [32], and for MacQueen’s bustard (home ranges of 116–977 km^2^, core areas of 13–128 km^2^ [142, 151].

## Conclusions

Male and female breeding home ranges were small during the breeding season, allowing for a high density of houbaras in the study area. During the breeding season both sexes selected non-cultivated habitats with low shrub coverage as foraging grounds, avoiding high density shrubland. During the non-breeding season *Launaea arborescens* shrubland continued to be the main foraging habitat, but both sexes spent a significant amount of time foraging on cultivated fields, and notably selected irrigated farmland (18% foraging locations in males, 27% in females). These irrigated fields seem to be of considerable value for the survival of houbaras during the driest months of the year, when natural resources in shrublands and pastures are scarcer. However, these cultivated fields need to be close enough to high density shrubland areas, the major foraging ground during that season, in order to be accessible to houbaras during their daily foraging routines. Thus, an appropriate mixture of shrubland, green fallows and a few irrigated fields seem to be the best habitat for houbaras in that season.

Finally, our study shows how Canarian houbaras, originally a desert-dwelling species, have developed the necessary adaptations to benefit from resources provided by current farmland conditions in our study area. Maintaining current habitat conditions in Lanzarote, i.e. a mosaic of *Launaea* shrubland with adequate amounts of fallows and irrigated cultivated fields, and improving those in Fuerteventura, should be prioritized as management measures in order to safeguard the future of this endangered species.

## Supplementary Information

**Additional file 1**. Supplementary tables and figures of Factors modulating home range and resource use: a case study with Canarian houbara bustards

## Data Availability

The data that support the findings of this study are available from Red Eléctrica de España. Restrictions apply to the availability of these data, which were used under license for this study. Data are also available from the authors with the permission from Red Eléctrica de España.

## Abbreviations

GLMMs: Generalized linear mixed models
KDE: Kernel density estimator
NDVI: Normalized difference vegetation index
SAVI: Soil-adjusted vegetation index
