# Supplementary material for: Factors modulating home range and resource use: a case study with Canarian houbara bustards
Source: Mov Ecol. 2022 Nov 14;10:49. doi: 10.1186/s40462-022-00346-1 (PMC9664789; doi:10.1186/s40462-022-00346-1)
Supplement: Supplementary file 1 — Additional file 1. Supplementary tables and figures of Factors modulating home range and resource use: a case study with Canarian houbara bustards [file 40462_2022_346_MOESM1_ESM.docx]

**Additional file 1. Supplemental tables**

**TABLE S1** Monthly home-range sizes of houbara bustards in Lanzarote (Canary Islands). Values are in km^2^.

|  |  | **K95** | | | | | **K50** | | | | | **MCP98** | | | |
| --- | --- | --- | --- | --- | --- | --- | --- | --- | --- | --- | --- | --- | --- | --- | --- |
|  |  | **** | **SD** | **min** | **max** |  | **** | **SD** | **min** | **max** |  | **** | **SD** | **min** | **max** |
| **Males n=22** | January | 0.72 | 0.92 | 0.19 | 3.53 |  | 0.11 | 0.20 | 0.02 | 0.80 |  | 1.42 | 2.14 | 0.35 | 8.58 |
|  | February | 0.80 | 0.97 | 0.22 | 3.40 |  | 0.12 | 0.18 | 0.02 | 0.74 |  | 0.97 | 0.85 | 0.37 | 3.63 |
|  | March | 0.82 | 0.91 | 0.23 | 3.93 |  | 0.14 | 0.17 | 0.04 | 0.74 |  | 1.27 | 1.25 | 0.32 | 5.43 |
|  | April | 0.98 | 0.76 | 0.25 | 2.86 |  | 0.17 | 0.14 | 0.05 | 0.50 |  | 1.59 | 1.24 | 0.38 | 4.68 |
|  | May | 0.99 | 0.92 | 0.22 | 3.64 |  | 0.17 | 0.14 | 0.03 | 0.50 |  | 1.48 | 1.40 | 0.27 | 5.41 |
|  | June | 0.96 | 0.47 | 0.26 | 2.18 |  | 0.18 | 0.09 | 0.05 | 0.42 |  | 1.52 | 0.81 | 0.38 | 3.45 |
|  | July | 0.95 | 1.12 | 0.18 | 3.50 |  | 0.18 | 0.23 | 0.03 | 0.80 |  | 1.08 | 0.91 | 0.23 | 3.66 |
|  | August | 0.76 | 0.67 | 0.27 | 3.29 |  | 0.13 | 0.09 | 0.04 | 0.38 |  | 1.00 | 0.82 | 0.41 | 3.83 |
|  | September | 0.78 | 0.45 | 0.26 | 2.07 |  | 0.15 | 0.08 | 0.04 | 0.35 |  | 1.46 | 1.50 | 0.36 | 6.08 |
|  | October | 1.45 | 1.42 | 0.26 | 5.34 |  | 0.24 | 0.21 | 0.05 | 0.88 |  | 1.99 | 2.25 | 0.37 | 8.72 |
|  | November | 1.53 | 1.19 | 0.45 | 4.18 |  | 0.29 | 0.25 | 0.09 | 0.99 |  | 1.95 | 1.34 | 0.63 | 4.59 |
|  | December | 1.12 | 1.20 | 0.22 | 3.85 |  | 0.18 | 0.23 | 0.02 | 0.81 |  | 1.78 | 1.69 | 0.32 | 5.26 |
| **Females n=21** | January | 1.18 | 0.74 | 0.31 | 2.27 |  | 0.82 | 1.26 | 0.04 | 1.76 |  | 1.43 | 0.88 | 0.39 | 3.06 |
|  | February | 1.43 | 1.44 | 0.27 | 4.67 |  | 0.26 | 0.32 | 0.02 | 1.06 |  | 1.91 | 1.56 | 0.31 | 4.88 |
|  | March | 1.19 | 0.38 | 0.63 | 1.53 |  | 1.11 | 1.65 | 0.09 | 1.35 |  | 2.33 | 0.79 | 1.05 | 3.24 |
|  | April | 1.14 | 0.81 | 0.27 | 2.44 |  | 0.20 | 0.14 | 0.04 | 0.38 |  | 1.72 | 1.42 | 0.46 | 4.62 |
|  | May | 0.53 | 0.29 | 0.24 | 1.81 |  | 0.38 | 0.52 | 0.04 | 1.24 |  | 1.95 | 2.58 | 0.41 | 7.71 |
|  | June | 1.05 | 0.71 | 0.25 | 2.27 |  | 0.36 | 0.52 | 0.04 | 1.88 |  | 1.85 | 1.40 | 0.46 | 4.93 |
|  | July | 0.78 | 0.78 | 0.14 | 3.12 |  | 0.16 | 0.15 | 0.02 | 0.63 |  | 1.36 | 1.44 | 0.30 | 5.54 |
|  | August | 0.93 | 0.84 | 0.18 | 3.34 |  | 0.32 | 0.40 | 0.04 | 1.36 |  | 1.15 | 0.70 | 0.31 | 2.97 |
|  | September | 0.57 | 0.32 | 0.14 | 0.94 |  | 0.11 | 0.06 | 0.03 | 0.18 |  | 1.14 | 0.83 | 0.27 | 2.96 |
|  | October | 1.34 | 1.50 | 0.28 | 5.68 |  | 0.23 | 0.19 | 0.04 | 0.74 |  | 2.29 | 2.67 | 0.59 | 9.81 |
|  | November | 2.00 | 1.88 | 0.39 | 8.10 |  | 0.71 | 0.97 | 0.06 | 3.12 |  | 2.79 | 2.31 | 0.80 | 10.59 |
|  | December | 1.60 | 1.26 | 0.18 | 4.68 |  | 0.39 | 0.32 | 0.03 | 1.12 |  | 1.91 | 1.53 | 0.40 | 5.69 |

**TABLE S2** Generalized linear mixed models explaining home range size (K95) in male houbara bustards.

| Covariate | Model | Parameter estimate | Lower CI | | Upper CI | Adjusted  SE | Z | P |
| --- | --- | --- | --- | --- | --- | --- | --- | --- |
| Intercept | S + P + S * P + RS + BW + HQ | 16.027 | 14.458 | 17.612 | | 0.753 | 21.280 | <0.001 |
|  | S + P + S * P + RS + BS + BW + HQ | 16.289 | 13.500 | 20.443 | | 1.144 | 14.233 | <0.001 |
|  | S + P + S * P + RS + BW | 16.035 | 14.460 | 17.630 | | 0.754 | 21.307 | <0.001 |
|  | S + P + S * P + RS +DF + DM + BS + BW + HQ | 16.162 | 13.282 | 20.269 | | 1.187 | 13.617 | <0.001 |
|  | S + P + S * P + RS + DM + BS + BW + HQ | 16.304 | 13.536 | 20.467 | | 1.131 | 14.412 | <0.001 |
|  | S + P + S * P + RS + DF + BS + BW + HQ | 16.180 | 13.402 | 20.383 | | 1.186 | 13.638 | <0.001 |
|  | S + P + S * P + RS + DF + BW | 15.836 | 14.196 | 17.494 | | 0.771 | 20.613 | <0.001 |
| Non-breeding season | S + P + S * P + RS + BW + HQ | -1.665 | -2.285 | -1.125 | | 0.302 | 5.515 | <0.001 |
|  | S + P + S * P + RS + BS + BW + HQ | -1.661 | -2.272 | -1.110 | | 0.302 | 5.501 | <0.001 |
|  | S + P + S * P + RS + BW | -1.522 | -2.088 | -0.965 | | 0.282 | 5.431 | <0.001 |
|  | S + P + S * P + RS +DF + DM + BS + BW + HQ | -1.657 | -2.272 | -1.092 | | 0.306 | 5.422 | <0.001 |
|  | S + P + S * P + RS + DM + BS + BW + HQ | -1.675 | -2.315 | -1.138 | | 0.303 | 5.524 | <0.001 |
|  | S + P + S * P + RS + DF + BS + BW + HQ | -1.650 | -2.252 | -1.071 | | 0.304 | 5.431 | <0.001 |
|  | S + P + S * P + RS + DF + BW | -1.485 | -2.056 | -0.923 | | 0.823 | 5.259 | <0.001 |
| Non-displaying  reproductive status | S + P + S * P + RS + BW + HQ | 1.795 | 1.409 | 2.234 | | 0.209 | 8.557 | <0.001 |
|  | S + P + S * P + RS + BS + BW + HQ | 1.793 | 1.400 | 2.226 | | 0.210 | 8.547 | <0.001 |
|  | S + P + S * P + RS + BW | 1.769 | 1.375 | 2.203 | | 0.210 | 8.451 | <0.001 |
|  | S + P + S * P + RS +DF + DM + BS + BW + HQ | 1.793 | 1.402 | 2.226 | | 0.209 | 8.542 | <0.001 |
|  | S + P + S * P + RS + DM + BS + BW + HQ | 1.792 | 1.395 | 2.220 | | 0.210 | 8.543 | <0.001 |
|  | S + P + S * P + RS + DF + BS + BW + HQ | 1.794 | 1.405 | 2.230 | | 0.210 | 8.548 | <0.001 |
|  | S + P + S * P + RS + DF + BW | 1.779 | 1.385 | 2.213 | | 0.210 | 8.503 | <0.001 |
| Non-breeding reproductive status | S + P + S * P + RS + BW + HQ | 1.835 | 1.253 | 2.400 | | 0.289 | 6.347 | <0.001 |
|  | S + P + S * P + RS + BS + BW + HQ | 1.830 | 1.230 | 2.383 | | 0.290 | 6.316 | <0.001 |
|  | S + P + S * P + RS + BW | 1.885 | 1.314 | 2.464 | | 0.289 | 6.540 | <0.001 |
|  | S + P + S * P + RS +DF + DM + BS + BW + HQ | 1.825 | 1.246 | 2.398 | | 0.293 | 6.236 | <0.001 |
|  | S + P + S * P + RS + DM + BS + BW + HQ | 1.829 | 1.233 | 2.386 | | 0.290 | 6.317 | <0.001 |
|  | S + P + S * P + RS + DF + BS + BW + HQ | 1.822 | 1.232 | 2.385 | | 0.293 | 6.219 | <0.001 |
|  | S + P + S * P + RS + DF + BW | 1.885 | 1.314 | 2.464 | | 0.289 | 6.547 | <0.001 |
| Precipitation | S + P + S * P + RS + BW + HQ | -0.017 | -0.028 | -0.007 | | 0.005 | 2.993 | 0.003 |
|  | S + P + S * P + RS + BS + BW + HQ | -0.017 | -0.027 | -0.007 | | 0.006 | 2.991 | 0.003 |
|  | S + P + S * P + RS + BW | -0.013 | -0.022 | -0.003 | | 0.004 | 2.682 | 0.007 |
|  | S + P + S * P + RS +DF + DM + BS + BW + HQ | -0.017 | -0.027 | -0.006 | | 0.006 | 2.967 | 0.003 |
|  | S + P + S * P + RS + DM + BS + BW + HQ | -0.017 | -0.027 | -0.007 | | 0.005 | 3.010 | 0.002 |
|  | S + P + S * P + RS + DF + BS + BW + HQ | -0.017 | -0.027 | -0.007 | | 0.006 | 3.000 | 0.002 |
|  | S + P + S * P + RS + DF + BW | -0.013 | -0.022 | -0.003 | | 0.006 | 2.685 | 0.007 |
| Season * Precipitation | S + P + S * P + RS + BW + HQ | 0.036 | 0.021 | 0.054 | | 0.008 | 4.431 | <0.001 |
|  | S + P + S * P + RS + BS + BW + HQ | 0.036 | 0.021 | 0.054 | | 0.008 | 4.440 | <0.001 |
|  | S + P + S * P + RS + BW | 0.033 | 0.017 | 0.049 | | 0.008 | 4.213 | <0.001 |
|  | S + P + S * P + RS +DF + DM + BS + BW + HQ | 0.036 | 0.020 | 0.053 | | 0.008 | 4.416 | <0.001 |
|  | S + P + S * P + RS + DM + BS + BW + HQ | 0.036 | 0.020 | 0.054 | | 0.008 | 4.440 | <0.001 |
|  | S + P + S * P + RS + DF + BS + BW + HQ | 0.036 | 0.021 | 0.054 | | 0.008 | 4.453 | <0.001 |
|  | S + P + S * P + RS + DF + BW | 0.033 | 0.017 | 0.050 | | 0.008 | 4.255 | <0.001 |
| Density of females | S + P + S * P + RS +DF + DM + BS + BW + HQ | 0.066 | -0.035 | 0.216 | | 0.065 | 1.015 | 0.310 |
|  | S + P + S * P + RS + DF + BS + BW + HQ | 0.027 | -0.068 | 0.118 | | 0.046 | 0.592 | 0.553 |
|  | S + P + S * P + RS + DF + BW | 0.040 | -0.053 | 0.133 | | 0.045 | 0.885 | 0.376 |
| Density of males | S + P + S * P + RS +DF + DM + BS + BW + HQ | -0.099 | -0.317 | 0.040 | | 0.091 | 1.079 | 0.280 |
|  | S + P + S * P + RS + DM + BS + BW + HQ | -0.050 | -0.182 | 0.081 | | 0.067 | 0.751 | 0.452 |
| Body size | S + P + S * P + RS + BS + BW + HQ | -0.002 | -0.011 | 0.006 | | 0.004 | 0.589 | 0.556 |
|  | S + P + S * P + RS + DF+ DM+ BS + BW + HQ | -0.002 | -0.001 | 0.006 | | 0.004 | 0.588 | 0.556 |
|  | S + P + S * P + RS + DM+ BS + BW + HQ | -0.002 | -0.011 | 0.006 | | 0.004 | 0.585 | 0.558 |
|  | S + P + S * P + RS + DF + BS + BW + HQ | -0.002 | -0.011 | 0.006 | | 0.004 | 0.599 | 0.549 |
| Body weight | S + P + S * P + RS + BW + HQ | -0.001 | -0.002 | -0.000 | | 0.000 | 3.023 | 0.002 |
|  | S + P + S * P + RS + BS + BW + HQ | -0.001 | -0.002 | -0.000 | | 0.000 | 3.006 | 0.002 |
|  | S + P + S * P + RS + BW | -0.001 | -0.002 | -0.000 | | 0.000 | 3.565 | 0.000 |
|  | S + P + S * P + RS +DF + DM + BS + BW + HQ | -0.001 | -0.002 | -0.000 | | 0.000 | 2.946 | 0.003 |
|  | S + P + S * P + RS + DM + BS + BW + HQ | -0.001 | -0.002 | -0.000 | | 0.000 | 3.009 | 0.003 |
|  | S + P + S * P + RS + DF + BS + BW + HQ | -0.001 | -0.002 | -0.000 | | 0.000 | 2.981 | 0.002 |
|  | S + P + S * P + RS + DF + BW | -0.001 | -0.002 | -0.000 | | 0.000 | 3.383 | 0.001 |
| Habitat quality | S + P + S * P + RS + BW + HQ | -3.178 | -5.894 | -0.365 | | 1.403 | 2.265 | 0.023 |
|  | S + P + S * P + RS + BS + BW + HQ | -3.187 | -5.911 | -0.406 | | 1.409 | 2.262 | 0.023 |
|  | S + P + S * P + RS +DF + DM + BS + BW + HQ | -3.195 | -5.829 | -0.228 | | 1.423 | 2.245 | 0.025 |
|  | S + P + S * P + RS + DM + BS + BW + HQ | -3.222 | -6.019 | -0.503 | | 1.411 | 2.283 | 0.022 |
|  | S + P + S * P + RS + DF + BS + BW + HQ | -3.186 | -5.835 | -0.251 | | 1.422 | 2.241 | 0.025 |

Model averaging. Parameter estimates, confidence intervals (CI), standard error and significance of generalized linear mixed models are shown. Only models with > 5% of the weight of evidence are shown (see Table 2). Season (S), reproductive status (RS), precipitation (P), density of females (DF), density of males (DM), body size (BS), body weight (BW) and habitat quality (HQ)

**TABLE S3** Generalized linear mixed models explaining home range size (K95) in female houbara bustards.

|  | Model | Parameter estimate | Lower CI | Upper CI | Adjusted SE | Z | P |
| --- | --- | --- | --- | --- | --- | --- | --- |
| Intercept | RS + P + DM + BS + BW + HQ | 3.033 | -2.890 | 8.955 | 3.022 | 1.004 | 0.315 |
|  | RS + P + DF + BS + BW + HQ | 4.844 | -0.861 | 10.551 | 2.912 | 1.664 | 0.096 |
|  | RS + P + DF + DM + BS + BW + HQ | 3.778 | -2.486 | 10.042 | 3.196 | 1.182 | 0.237 |
|  | S + RS + P + DM + BS + BW + HQ | 3.273 | -2.715 | 9.262 | 3.055 | 1.071 | 0.284 |
|  | S + RS + P + DF + BS + BW + HQ | 4.951 | -0.823 | 10.726 | 2.946 | 1.681 | 0.093 |
|  | S + RS + P + DF + DM + BS + BW + HQ | 3.871 | -2.345 | 10.088 | 3.171 | 1.221 | 0.222 |
|  | RS + DF + BS + BW + HQ | 5.376 | -0.364 | 11.115 | 9.928 | 1.836 | 0.066 |
|  | S + P + DF + BS + BW + HQ | 6.846 | -1.205 | 12.486 | 2.878 | 2.379 | 0.017 |
| Non-breeding season | S + RS + P + DM + BS + BW + HQ | 0.711 | -0.373 | 1.795 | 0.553 | 1.285 | 0.199 |
|  | S + RS + P + DF + BS + BW + HQ | 0.412 | -0.876 | 1.700 | 0.657 | 0.627 | 0.531 |
|  | S + RS + P + DF + DM + BS + BW + HQ | 0.621 | -0.597 | 1.809 | 0.606 | 1.024 | 0.306 |
|  | S + P + DF + BS + BW + HQ | 0.572 | -0.050 | 1.194 | 0.317 | 1.803 | 0.071 |
| Mating  reproductive status | RS + P + DM + BS + BW + HQ | 1.087 | -0.285 | 2.458 | 0.699 | 1.552 | 0.120 |
|  | RS + P + DF + BS + BW + HQ | 1.412 | -0.028 | 2.797 | 0.706 | 2.000 | 0.045 |
|  | RS + P + DF + DM + BS + BW + HQ | 1.242 | -0.028 | 2.793 | 0.722 | 1.721 | 0.085 |
|  | S + RS + P + DM + BS + BW + HQ | 1.084 | -0.283 | 2.452 | 0.698 | 1.554 | 0.120 |
|  | S + RS + P + DF + BS + BW + HQ | 1.411 | -0.028 | 2.793 | 0.705 | 2.00 | 0.045 |
|  | S + RS + P + DF + DM + BS + BW + HQ | 1.237 | -0.177 | 2.651 | 0.721 | 1.714 | 0.086 |
|  | RS + DF + BS + BW + HQ | 1.284 | -0.091 | 2.659 | 0.702 | 1.830 | 0.067 |
| Nesting  reproductive status | RS + P + DM + BS + BW + HQ | 0.242 | -1.396 | 1.881 | 0.836 | 0.290 | 0.772 |
|  | RS + P + DF + BS + BW + HQ | 0.386 | -1.252 | 2.023 | 0.835 | 0.462 | 0.644 |
|  | RS + P + DF + DM + BS + BW + HQ | 0.309 | -1.335 | 1.955 | 0.839 | 0.369 | 0.712 |
|  | S + RS + P + DM + BS + BW + HQ | 0.252 | -1.387 | 1.891 | 0.836 | 0.301 | 0.763 |
|  | S + RS + P + DF + BS + BW + HQ | 0.389 | -1.248 | 2.028 | 0.836 | 0.466 | 0.641 |
|  | S + RS + P + DF + DM + BS + BW + HQ | 0.314 | -1.331 | 1.959 | 0.839 | 0.374 | 0.708 |
|  | RS + DF + BS + BW + HQ | 0.215 | -1.413 | 1.844 | 0.831 | 0.259 | 0.795 |
| Not breeding  reproductive status | RS + P + DM + BS + BW + HQ | 1.554 | 0.379 | 2.728 | 0.599 | 2.594 | 0.009 |
|  | RS + P + DF + BS + BW + HQ | 1.854 | 0.652 | 3.056 | 0.613 | 3.023 | 0.002 |
|  | RS + P + DF + DM + BS + BW + HQ | 1.706 | 0.485 | 2.928 | 0.623 | 2.738 | 0.006 |
|  | S + RS + P + DM + BS + BW + HQ | 1.550 | 0.382 | 2.724 | 0.598 | 2.600 | 0.009 |
|  | S + RS + P + DF + BS + BW + HQ | 1.851 | 0.651 | 3.051 | 0.612 | 3.023 | 0.002 |
|  | S + RS + P + DF + DM + BS + BW + HQ | 1.701 | 0.481 | 2.921 | 0.622 | 2.733 | 0.006 |
|  | RS + DF + BS + BW + HQ | 1.829 | 0.629 | 3.029 | 0.912 | 2.989 | 0.003 |
| Non-reproductive status | RS + P + DM + BS + BW + HQ | 1.656 | 0.487 | 2.825 | 0.596 | 2.777 | 0.005 |
|  | RS + P + DF + BS + BW + HQ | 1.699 | 0.529 | 2.870 | 0.597 | 2.847 | 0.004 |
|  | RS + P + DF + DM + BS + BW + HQ | 1.686 | 0.516 | 2.855 | 0.596 | 2.826 | 0.005 |
|  | S + RS + P + DM + BS + BW + HQ | 1.466 | 0.001 | 2.931 | 0.748 | 1.961 | 0.050 |
|  | S + RS + P + DF + BS + BW + HQ | 1.624 | 0.267 | 2.981 | 0.692 | 2.346 | 0.019 |
|  | S + RS + P + DF + DM + BS + BW + HQ | 1.545 | 0.123 | 2.968 | 0.726 | 2.129 | 0.033 |
|  | RS + DF + BS + BW + HQ | 1.546 | 0.403 | 2.687 | 0.582 | 2.652 | 0.008 |
| Precipitation | RS + P + DM + BS + BW + HQ | 0.025 | 0.005 | 0.044 | 0.010 | 2.443 | 0.014 |
|  | RS + P + DF + BS + BW + HQ | 0.025 | 0.005 | 0.045 | 0.010 | 2.502 | 0.012 |
|  | RS + P + DF + DM + BS + BW + HQ | 0.025 | 0.005 | 0.045 | 0.010 | 2.464 | 0.013 |
|  | S + RS + P + DM + BS + BW + HQ | 0.026 | 0.006 | 0.047 | 0.010 | 2.559 | 0.010 |
|  | S + RS + P + DF + BS + BW + HQ | 0.026 | 0.006 | 0.046 | 0.010 | 2.538 | 0.011 |
|  | S + RS + P + DF + DM + BS + BW + HQ | 0.026 | 0.006 | 0.046 | 0.010 | 2.541 | 0.011 |
|  | S + P + DF + BS + BW + HQ | 0.028 | 0.008 | 0.049 | 0.010 | 2.682 | 0.007 |
| Density of females | RS + P + DF + BS + BW + HQ | -0.360 | -0.569 | -0.152 | 0.106 | 3.385 | 0.001 |
|  | RS + P + DF + DM + BS + BW + HQ | -0.299 | -0.613 | 0.014 | 0.160 | 1.870 | 0.064 |
|  | S + RS + P + DF + BS + BW + HQ | -0.352 | -0.567 | -0.137 | 0.110 | 3.206 | 0.001 |
|  | S + RS + P + DF + DM + BS + BW + HQ | -0.274 | -0.619 | 0.071 | 0.176 | 1.556 | 0.119 |
|  | RS + DF + BS + BW + HQ | -0.373 | -0.586 | -0.159 | 0.109 | 3.419 | 0.001 |
|  | S + P + DF + BS + BW + HQ | -0.261 | -0.453 | -0.069 | 0.097 | 2.673 | 0.007 |
| Density of males | RS + P + DM + BS + BW + HQ | -0.434 | -0.685 | -0.183 | 0.128 | 3.338 | 0.001 |
|  | RS + P + DF + DM + BS + BW + HQ | -0.372 | -0.740 | -0.004 | 0.188 | 1.983 | 0.047 |
|  | S + RS + P + DM + BS + BW + HQ | -0.423 | -0.678 | -0.168 | 0.130 | 3.251 | 0.001 |
|  | S + RS + P + DF + DM + BS + BW + HQ | -0.377 | -0.728 | -0.026 | 0.179 | 2.104 | 0.035 |
| Body size | RS + P + DM + BS + BW + HQ | -0.025 | -0.042 | -0.008 | 0.009 | 2.943 | 0.003 |
|  | RS + P + DF + BS + BW + HQ | -0.030 | -0.046 | -0.014 | 0.008 | 3.689 | <0.001 |
|  | RS + P + DF + DM + BS + BW + HQ | -0.027 | -0.045 | -0.010 | 0.009 | 3.141 | 0.002 |
|  | S + RS + P + DM + BS + BW + HQ | -0.026 | -0.044 | -0.009 | 0.009 | 2.997 | 0.003 |
|  | S + RS + P + DF + BS + BW + HQ | -0.031 | -0.047 | -0.014 | 0.008 | 3.695 | <0.001 |
|  | S + RS + P + DF + DM + BS + BW + HQ | -0.028 | -0.045 | -0.010 | 0.009 | 3.159 | 0.001 |
|  | RS + DF + BS + BW + HQ | -0.030 | -0.046 | -0.014 | 0.008 | 3.642 | <0.001 |
|  | S + P + DF + BS + BW + HQ | -0.034 | -0.051 | -0.018 | 0.008 | 4.060 | <0.001 |
| Body weight | RS + P + DM + BS + BW + HQ | 0.004 | 0.002 | 0.005 | 0.001 | 3.816 | 0.001 |
|  | RS + P + DF + BS + BW + HQ | 0.003 | 0.001 | 0.005 | 0.001 | 3.501 | <0.001 |
|  | RS + P + DF + DM + BS + BW + HQ | 0.003 | 0.001 | 0.005 | 0.001 | 3.602 | <0.001 |
|  | S + RS + P + DM + BS + BW + HQ | 0.004 | 0.002 | 0.006 | 0.001 | 3.872 | <0.001 |
|  | S + RS + P + DF + BS + BW + HQ | 0.003 | 0.001 | 0.005 | 0.001 | 3.507 | <0.001 |
|  | S + RS + P + DF + DM + BS + BW + HQ | 0.003 | 0.002 | 0.005 | 0.001 | 3.645 | <0.001 |
|  | RS + DF + BS + BW + HQ | 0.003 | 0.001 | 0.005 | 0.001 | 3.282 | 0.001 |
|  | S + P + DF + BS + BW + HQ | 0.004 | 0.001 | 0.005 | 0.001 | 3.494 | 0.001 |
| Habitat quality | RS + P + DM + BS + BW + HQ | 10.014 | 3.379 | 16.648 | 3.385 | 2.958 | 0.003 |
|  | RS + P + DF + BS + BW + HQ | 10.025 | 4.443 | 17.335 | 0.010 | 2.02 | 0.012 |
|  | RS + P + DF + DM + BS + BW + HQ | 10.435 | 3.832 | 17.038 | 3.369 | 3.097 | 0.002 |
|  | S + RS + P + DM + BS + BW + HQ | 11.240 | 3.832 | 18.653 | 3.780 | 2.973 | 0.003 |
|  | S + RS + P + DF + BS + BW + HQ | 11.310 | 4.484 | 18.137 | 3.483 | 3.247 | 0.001 |
|  | S + RS + P + DF + DM + BS + BW + HQ | 11.251 | 4.087 | 18.415 | 3.655 | 3.078 | 0.002 |
|  | RS + DF + BS + BW + HQ | 10.334 | 3.924 | 16.748 | 3.271 | 3.159 | 0.002 |
|  | S + P + DF + BS + BD + HQ | 12.486 | 5.110 | 19.861 | 3.763 | 3.318 | 0.001 |

Model averaging. Parameter estimates, confidence intervals (CI), standard error and significance of generalized linear mixed models are shown. Only models with > 5% of the weight of evidence are shown (see Table 3). Season (S), reproductive status (RS), precipitation (P), density of females (DF), density of males (DM), body size (BS), body weight (BW) and habitat quality (HQ)

**TABLE S4.** Percentage distributions among different habitat types of houbara foraging locations.

|  | Males (n=18) | | | |  | Females (n=19) | | | |  |
| --- | --- | --- | --- | --- | --- | --- | --- | --- | --- | --- |
| Habitat types | Breeding season | |  | Non-breeding season |  | Breeding season | |  | Non-breeding season |  |
|  | Displaying  n =16 | Not displaying  n =2 |  |  |  | Breeding  n =15 ^2^ | Not breeding  n = 4 |  |  |  |
|  |  |  |  |  |  |  |  |  |  |  |
| Pastures | 10.92 | 98.89 |  | 5.20 |  | 46.03 | 44.78 |  | 11.38 |  |
| High density shrubland | 3.29 | 0.00 |  | 40.71 |  | 4.70 | 0.03 |  | 11.79 |  |
| Low density shrubland | 81.11 | 0.37 |  | 31.90 |  | 41.56 | 10.70 |  | 39.28 |  |
| Sweet potato fallow | 0.02 | 0.74 |  | 2.01 |  | 0.14 | 10.70 |  | 0.61 |  |
| White fallow | 1.35 | 0.00 |  | 0.00 |  | 3.33 | 2.70 |  | 0.10 |  |
| Green fallow | 3.04 | 0.00 |  | 4.46 |  | 3.19 | 10.86 |  | 10.87 |  |
| Alfalfa | 0.00 | 0.00 |  | 0.00 |  | 0.00 | 0.00 |  | 3.09 |  |
| Orchards | 0.27 | 0.00 |  | 3.38 |  | 0.00 | 0.00 |  | 0.69 |  |
| Clean orchards | 0.00 | 0.00 |  | 0.54 |  | 0.00 | 0.00 |  | 1.91 |  |
| Sweet potato | 0.00 | 0.00 |  | 10.14 |  | 0.78 | 19.24 |  | 18.67 |  |
| Sweet potato/fallow | 0.00 | 0.00 |  | 1.67 |  | 0.27 | 0.99 |  | 1.61 |  |
|  |  |  |  |  |  |  |  |  |  |  |
| Non-cultivated^1^ | 99.71 | 99.26 |  | 82.27 |  | 98.81 | 69.07 |  | 73.42 |  |
| Cultivated^1^ | 0.29 | 0.74 |  | 17.73 |  | 1.19 | 30.93 |  | 26.58 |  |
|  |  |  |  |  |  |  |  |  |  |  |
| Irrigated | - ^3^ | - ^3^ |  | 84.54 |  | 40.39 | 48.84 |  | 64.10 |  |
| Not irrigated | - ^3^ | - ^3^ |  | 10.46 |  | 59.61 | 51.16 |  | 30.90 |  |
|  |  |  |  |  |  |  |  |  |  |  |
| No. locations | 5496 | 542 |  | 12992 |  | 17048 | 3747 |  | 18156 |  |

See Methods for definition of habitat types. ^1^ Non-cultivated land is the sum of pastures, high- and low-density *Launaea* shrubland, and green and white fallows, and cultivated land is the sum of sweet potato fallow, alfalfas, orchards, clean orchards, sweet potato fields and sweet potato/fallow-fields. ^2^ This sample includes foraging locations from 9 females in 2021; these 9 females could not be added in home range analyses (Tables 1 and 3) because SAVI values were not available for 2021. ^3^ Percentage use not calculated due to a very small sample size (displaying males: only 16 locations; not displaying males: only 4 locations; in both cases all in the same field, and corresponding to the same individual)

**TABLE S5** Generalized linear mixed models quantifying fine-scale resource selection of displaying male houbara bustards in the breeding season.

| Covariates | Parameter estimate | Adjusted SE | Z | P |
| --- | --- | --- | --- | --- |
| Pastures | -2.389 | 0.307 | 7.774 | <0.001 |
| High density shrubland | -3.079 | 0.313 | 9.828 | <0.001 |
| Low density shrubland | 1.177 | 0.296 | 3.939 | <0.001 |
| Sweet potato fallow | -4.242 | 1.050 | -4.039 | <0.001 |
| White fallow | -0.598 | 0.326 | 1.831 | 0.067 |
| Green fallow | 2.506 | 0.380 | 6.593 | <0.001 |
| Orchards | 3.072 | 1.733 | 0.002 | 0.998 |
| Clean orchards | -9.591 | 9.123 | 0.001 | 0.999 |
| Sweet potato | -3.864 | 1.000 | 0.004 | 0.996 |
| Sweet potato/fallow | -3.947 | 1.000 | 0.004 | 0.996 |

Model averaging. Parameter estimates, confidence intervals (CI), standard error and significance of generalized linear mixed models are shown.

**Table S6** Generalized linear mixed models quantifying fine-scale resource selection of male houbara bustards during the non-breeding season.

| Covariates | Parameter estimate | Adjusted SE | Z | P |
| --- | --- | --- | --- | --- |
| Pastures | -1.334 | 0.163 | 8.190 | <0.001 |
| High density shrubland | 2.245 | 0.159 | 14.069 | <0.001 |
| Low density shrubland | -0.254 | 0.155 | 1.640 | 0.101 |
| Sweet potato fallow | 24.007 | 8897.90 | 0.003 | 0.997 |
| White fallow | -23.872 | 12028.47 | -0.002 | 0.998 |
| Green fallow | 0.271 | 0.167 | 1.625 | 0.104 |
| Orchards | 0.467 | 0.171 | 2.731 | 0.006 |
| Clean orchards | -2.756 | 0.198 | -13.920 | <0.001 |
| Sweet potato | 1.512 | 0.166 | 9.093 | <0.001 |
| Sweet potato/fallow | -0.151 | 0.178 | 0.848 | 0.396 |

Model averaging. Parameter estimates, confidence intervals (CI), standard error and significance of generalized linear mixed models are shown.

**TABLE S7** Generalized linear mixed models quantifying fine-scale resource selection of breeding female houbara bustards in the breeding season.

| Covariates | Parameter estimate | Adjusted SE | Z | P |
| --- | --- | --- | --- | --- |
| Pastures | 0.554 | 0.098 | 5.635 | <0.001 |
| High density shrubland | -0.934 | 0.105 | 8.855 | <0.001 |
| Low density shrubland | -0.326 | 0.095 | 3.402 | <0.001 |
| Sweet potatofallow | 22.160 | 1205 | 0.002 | 0.998 |
| White fallow | 2.411 | 0.173 | 13.937 | <0.001 |
| Green fallow | 1.118 | 0.133 | 8.383 | <0.001 |
| Orchards | -20.73 | 8166 | 0.003 | 0.997 |
| Clean orchards | -34.94 | 3.391 | 0.000 | 0.999 |
| Sweet potato | 33.11 | 1.221 | 0.000 | 0.999 |
| Sweet potato/fallow | 0.536 | 0.246 | 2.177 | 0.029 |

Model averaging. Parameter estimates, confidence intervals (CI), standard error and significance of generalized linear mixed models are shown.

**TABLE S8** Generalized linear mixed models quantifying fine-scale resource selection of female houbara bustards during the non-breeding season.

| Covariates | Parameter estimate | Adjusted SE | Z | P |
| --- | --- | --- | --- | --- |
| Pastures | -0.179 | 0.061 | 2.939 | 0.003 |
| High density shrubland | 0.315 | 0.064 | 4.943 | <0.001 |
| Low density shrubland | 0.029 | 0.054 | 0.537 | 0.591 |
| Sweet potato fallow | -1.494 | 0.118 | 12.698 | <0.001 |
| White fallow | -4.306 | 0.245 | 17.582 | <0.001 |
| Green fallow | 0.266 | 0.062 | 4.305 | <0.001 |
| Alfalfa | 23.048 | 4490 | 0.005 | 0.996 |
| Orchards | -2.269 | 0.109 | 20.778 | <0.001 |
| Clean orchards | -0.771 | 0.087 | 8.824 | <0.001 |
| Sweet potato | 1.150 | 0.063 | 18.333 | <0.001 |
| Sweet potato/fallow | -1.083 | 0.086 | 12.548 | <0.001 |

Model averaging. Parameter estimates, confidence intervals (CI), standard error and significance of generalized linear mixed models are shown.

**Additional file 2. Supplemental figures**

**FIGURE S1** Home range (KDE95) of males (A) in the breeding season and females (B) in the non-breeding season.


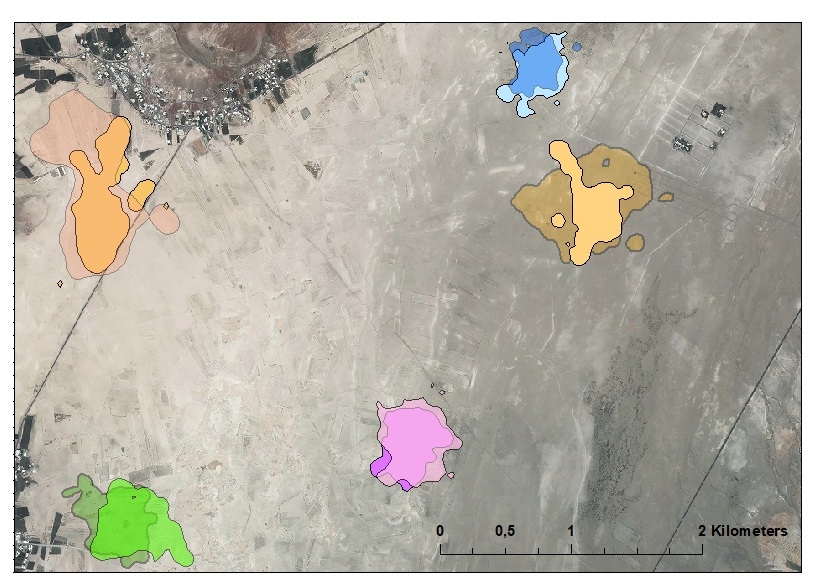


**A**


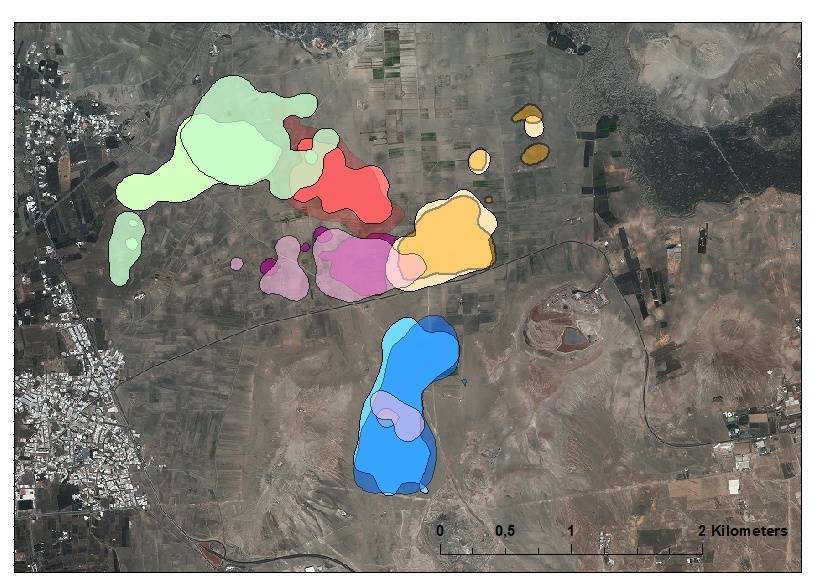


**B**

Each colour corresponds to a different individual, and the transparent and opaque areas correspond respectively to 2018 and 2019. The orthophoto was obtained from Centro Nacional de Información Geográfica (PNOA).

**FIGURE S2**. Changes in home-range estimates (95% KDE, km^2^) of female Canarian houbara bustards with reproductive status.


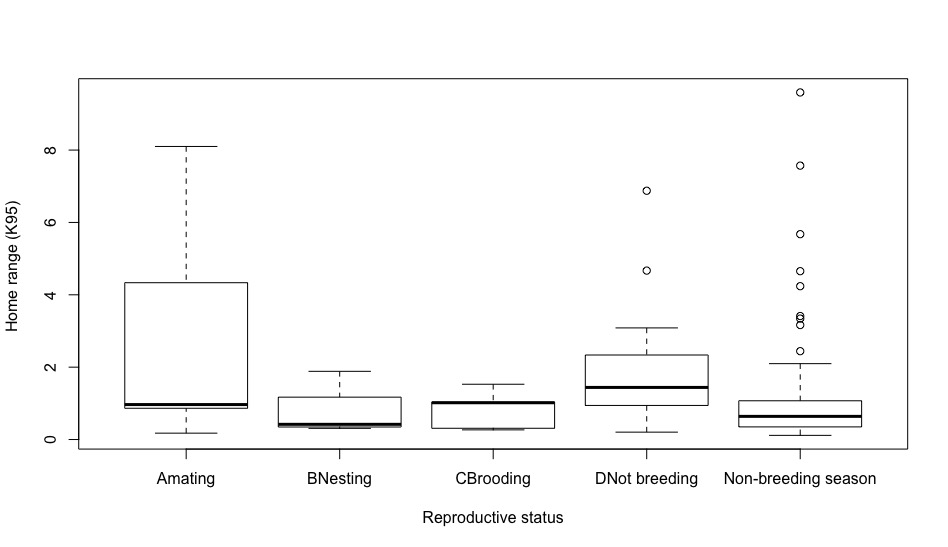


Mating Nesting Brooding Not breeding Non-breeding season

Reproductive status

Home range (K95)

Boxes contain the 25th to 75th percentiles, and thick lines show the medians. Whiskers show the minimum and maximum values, and dots show outlier values.
